# Supplementary material for: De novo protein-coding gene variants in developmental stuttering
Source: Mol Psychiatry. 2025 Aug 20;31(1):104–15. doi: 10.1038/s41380-025-03170-2 (PMC12700790; doi:10.1038/s41380-025-03170-2)
Supplement: Supplementary file 1 — Supplementary information to de novo protein-coding gene variants in developmental stuttering [file 41380_2025_3170_MOESM1_ESM.docx]

# Supplementary information to

# *De novo* protein-coding gene variants in developmental stuttering

Else Eising^1^*, Ivana Dzinovic^2,3^, Arianna Vino^1^, Lottie Stipdonk^4^, Martin Pavlov^2,3^, Juliane Winkelmann^2,3,5-7^, Martin Sommer^8,9^, Marie-Christine J.P. Franken^4^, Konrad Oexle^2,3,10,#^, Simon E. Fisher^1,11,#^

# Supplemental Figures


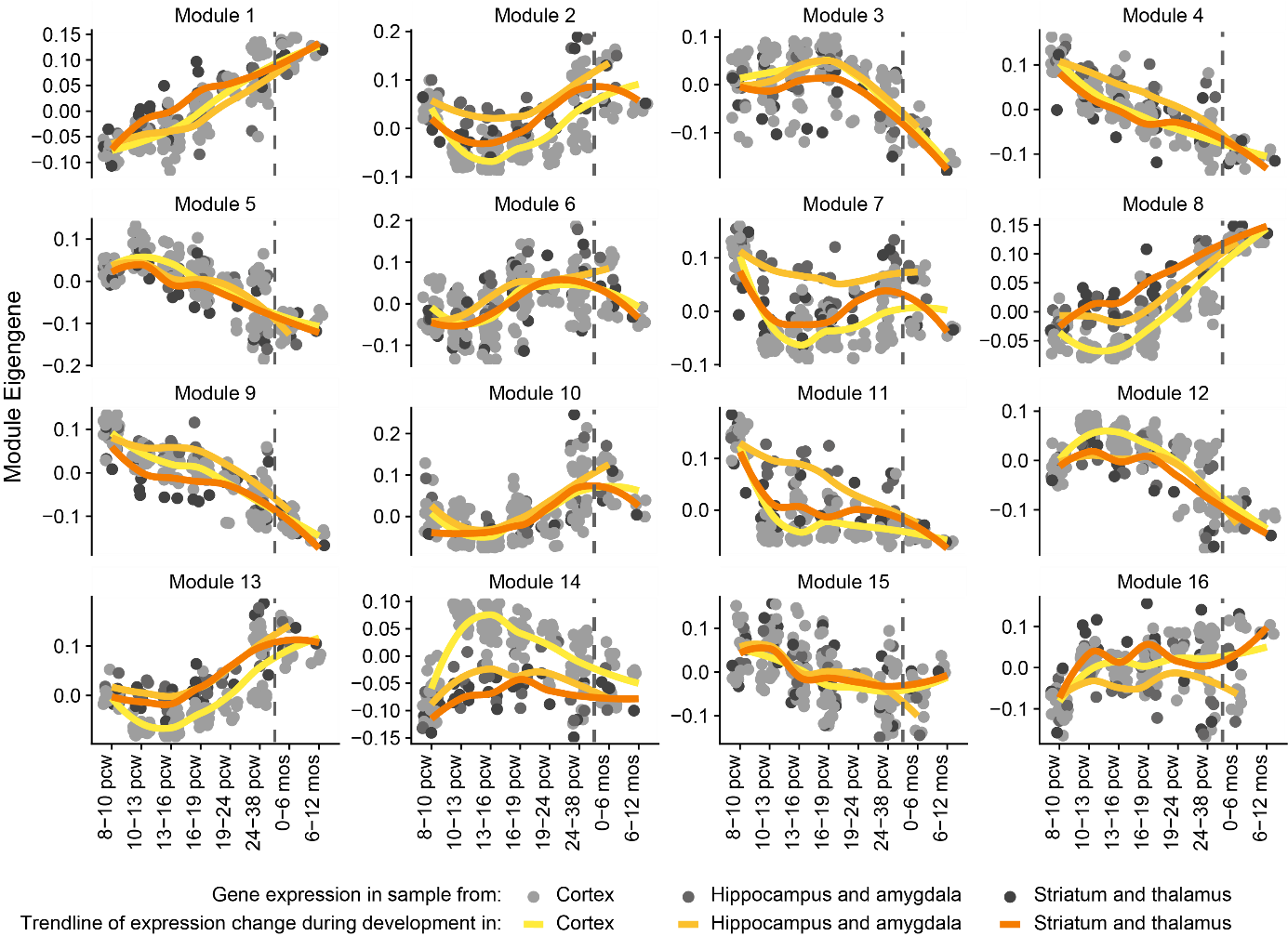


**Supplemental Figure 1: Expression pattern of gene expression modules during brain development.** Module Eigengenes represent the overlapping expression pattern of all genes represented by the module. Each dot represents a brain sample, the yellow lines are the loess curve ﬁtted through the data points. The vertical dashed lines represent time of birth. Pcw: post conception week.

# Supplemental Tables

**Supplemental Table 1: overview of participants**

| Proband | St | Sex | Age^@^ at participation | Age at onset (years) | Self-report speech fluency^#^ | Parent-report speech fluency^#^ | Therapist-report speech fluency | SSI | Trialled therapies | Family members who stutter(ed) | Other diagnoses |
| --- | --- | --- | --- | --- | --- | --- | --- | --- | --- | --- | --- |
| RESTART_1 | T | Male | 11-15 | 5 | Mild / mild to moderate | Borderline | - | 2 | Lidcombe program | No | Dyslexia |
| RESTART_2 | P | Male | 11-15 | 3 | Mild | Mild | Fluent | 11 | RESTART-DCM | No | Dyslexia |
| RESTART_3 | T | Female | 11-15 | 5 | Fluent | Fluent | Fluent | 6 | RESTART-DCM | 2nd degree relative | No |
| RESTART_4 | T | Male | 11-15 | 3 | Fluent | Fluent | Fluent | 2 | Lidcombe program | 4th degree relative | No |
| RESTART_5 | T | Male | 11-15 | 4 | Fluent | Fluent | Fluent | 6 | Lidcombe program | No | Dyslexia |
| RESTART_6 | P | Male | 11-15 | 3 | Borderline | Fluent | Fluent | 13 | Lidcombe program | No | No |
| RESTART_7 | P | Male | 11-15 | 4 | Mild to moderate | Mild | Mild | 12 | Lidcombe program | No | Dyslexia |
| RESTART_8 | A | Male | 11-15 | 5 | Mild | Mild | Fluent | 0 | Lidcombe program | No | Behaviour problems, social/emotional problems, autism |
| RESTART_9 | A | Female | 11-15 | 5 | Borderline | Fluent | Fluent / borderline | 12 | Lidcombe program | No | No |
| RESTART_10 | T | Male | 11-15 | 5 | Borderline | Fluent | Fluent | - | Lidcombe program | No | No |
| RESTART_11 | P | Male | 11-15 | 6 | Mild to moderate | Mild to moderate | Mild to moderate | 25 | RESTART-DCM | No | Social/emotional problems, hypermobility |
| RESTART_12 | T | Male | 11-15 | 4 | Fluent | Fluent | Mild | 2 | Lidcombe program | No | ADD/ADHD |
| RESTART_13 | T | Female | 11-15 | 4 | Borderline | Fluent | Fluent / borderline | 2 | Lidcombe program | 2nd and 4th degree relative | Social/emotional problems |
| RESTART_14 | A | Male | 6-10 | 3 | Mild to moderate | Fluent | Fluent / borderline | - | RESTART-DCM | No | No |
| RESTART_15 | P | Male | 11-15 | 4 | Moderate | Moderate | Mild | - | Lidcombe program | 2nd degree relative | No |
| RESTART_16 | T | Female | 11-15 | 4 | Borderline | Fluent | Fluent | - | Lidcombe program | No | No |
| RESTART_17 | T | Female | 11-15 | 4 | Fluent | Fluent | Fluent | 2 | RESTART-DCM | No | No |
| RESTART_18 | T | Male | 11-15 | 5 | Fluent | Fluent | Fluent | 8 | RESTART-DCM | 4th degree relative | No |
| RESTART_19 | P | Male | 6-10 | 3 | Fluent | Fluent | Fluent | 20 | RESTART-DCM | No | No |
| RESTART_20 | T | Male | 11-15 | 4 | Fluent | Fluent | Fluent | - | Lidcombe program | No | No |
| RESTART_21 | P | Male | 6-10 | 3 | Mild | Mild to moderate | Mild to moderate | 27 | RESTART-DCM | No | No |
| RESTART_22 | T | Male | 11-15 | 4 | Fluent | Fluent | Fluent | 3 | Lidcombe program | No | Low intelligence, social/emotional problems, autism, ADD/ADHD |
| RESTART_23 | P | Male | 6-10 | 3 | Fluent | Fluent | Borderline | 19 | Lidcombe program | Cousin of mother | No |
| RESTART_24 | P | Male | 11-15 | 6 | Mild to moderate / moderate | Mild to moderate | Moderate to severe | 32 | RESTART-DCM | Cousin of mother (2x) | No |
| RESTART_25 | P | Male | 11-15 | 5 | Mild to moderate | Mild / mild to moderate | Mild / mild to moderate | 26 | Lidcombe program | No | No |
| RESTART_26 | T | Male | 11-15 | 5 | Borderline | Fluent | Borderline | 2 | RESTART-DCM | 4th degree relative | No |
| RESTART_27 | T | Female | 6-10 | 4 | Borderline | Fluent | - | 2 | RESTART-DCM | 4th degree relative (2x) | No |
| RESTART_28 | T | Male | 6-10 | 3 | Fluent | Fluent | Fluent | 4 | Lidcombe program | No | No |
| RESTART_29 | T | Female | 11-15 | 4 | Borderline | Fluent | Borderline | 2 | RESTART-DCM | 3rd degree relative | No |
| RESTART_30 | T | Female | 6-10 | 3 | Fluent | Fluent | Fluent | 2 | Lidcombe program | 2nd degree relative | No |
| RESTART_31 | T | Female | 6-10 | 3 | Fluent | Fluent | Fluent | 2 | Lidcombe program | No | Hearing problems |
| RESTART_32 | P | Male | 11-15 | 4 | Mild to moderate | Mild | Mild | - | Lidcombe program | 3rd degree relative | Dyslexia |
| RESTART_33 | P | Male | 11-15 | 5 | Moderate | Moderate | Moderate to severe | 33 | RESTART-DCM | No | No |
| RESTART_34 | A | Male | 6-10 | 4 | Fluent | Fluent | Fluent | 8 | Lidcombe program | No | Dyslexia |
| RESTART_35 | T | Female | 11-15 | 6 | Fluent | Fluent | Borderline case | 2 | RESTART-DCM | No | Problems with speech and language |
| RESTART_36 | P | Male | 11-15 | 6 | Fluent | Fluent | Fluent / borderline | 15 | RESTART-DCM | No | ADD/ADHD |
| RESTART_37 | T | Female | 6-10 | 3 | Fluent | Fluent | Fluent | 0 | Lidcombe program | No | No |
| RESTART_38 | T | Male | 6-10 | 3 | Fluent | Fluent | Fluent | - | RESTART-DCM | No | No |
| RESTART_39 | T | Male | 6-10 | 4 | Fluent | Fluent | Fluent | 2 | Lidcombe program | No | No |
| RESTART_40 | T | Female | 6-10 | 4 | Fluent | Fluent | Fluent | 0 | RESTART-DCM | 2nd and 4th degree relative | No |
| RESTART_41 | T | Male | 11-15 | 4 | Fluent | Fluent | - | - | Lidcombe program | 4th degree relative | Dyslexia, ADD/ADHD |
| RESTART_42 | P | Male | 11-15 | 6 | Moderate | Mild to moderate | Moderate | 28 | RESTART-DCM | 2nd and 3rd degree relative | ADD/ADHD, Gilles de la Tourette |
| RESTART_43 | T | Male | 6-10 | 3 | Fluent | Fluent | Borderline | - | Lidcombe program | 4th degree relative | No |
| RESTART_44 | T | Male | 6-10 | 3 | Borderline | Borderline | Borderline | 2 | Lidcombe program | 2nd and 3rd degree relative | No |
| RESTART_45 | A | Male | 10 | 5 | Borderline | Mild | Fluent | - | RESTART-DCM | No | Dyslexia |
| RESTART_46 | P | Male | 11-15 | 5 | Moderate | Moderate to severe | Moderate to severe | 32 | Lidcombe program | 3rd degree relative | No |
| RESTART_47 | P | Male | 11-15 | 6 | Moderate | Mild | Borderline | 7 | Lidcombe program | No | No |
| RESTART_48 | A | Male | 6-10 | 3 | Fluent | Fluent | Fluent | 2 | RESTART-DCM | No | Dyslexia, ADD/ADHD |
| RESTART_49 | T | Female | 6-10 | 3 | Fluent | Borderline | - | 2 | Lidcombe program | 2nd degree relative | Hearing problems |
| RESTART_50 | T | Male | 6-10 | 3 | Fluent | Fluent | Borderline | - | RESTART-DCM | 3rd degree relative | Dyslexia |
| RESTART_51 | T | Male | 6-10 | 3 | Fluent | Fluent | Fluent | 3 | RESTART-DCM | No | Dyslexia |
| RESTART_52 | T | Female | 6-10 | 4 | Fluent | Fluent | Fluent | 2 | RESTART-DCM | 2nd degree relative | ADD/ADHD |
| RESTART_53 | P | Male | 6-10 | 5 | Mild to moderate | Moderate | Moderate | 28 | Lidcombe program | 2nd degree relative | Dyslexia, lateralization problems |
| RESTART_54 | P | Male | 6-10 | 4 | Mild | Mild to moderate | Mild | 24 | RESTART-DCM | 4th degree relative | Hearing problems |
| RESTART_55 | A | Male | 11-15 | 6 | Mild to moderate | Moderate | Mild | 24 | RESTART-DCM | 4th degree relative | No |
| RESTART_56 | P | Male | 6-10 | 4 | Borderline | Fluent | Fluent / borderline | 23 | Lidcombe program, speech/stutter therapy (unknown method) | No | No |
| RESTART_57 | A | Male | 6-10 | 4 | Severe | Fluent | Mild | - | RESTART-DCM | No | Dyslexia |
| MEGS_1 | P | Female | 6-10 | 5 to 6 | - | 5 | - | - | Speech/stutter therapy: (unknown method) + to learn speech techniques + to change negative thoughts and feelings | No | No |
| MEGS_2 | P | Male | 6-10 | 1 to 2 | - | 7 | - | - | Speech/stutter therapy to learn speech techniques | Na | No |
| MEGS_3 | P | Male | 6-10 | 3 | - | 8 | - | - | Speech/stutter therapy (unknown method); | 2nd degree relative | No |
| MEGS_4 | P | Male | 6-10 | 1 to 2 | - | 2 | - | - | Speech/stutter therapy: (unknown method) + to learn speech techniques | No | No |
| MEGS_5 | P | Male | 11-15 | 1 to 2 | - | 5 | - | - | Lidcombe program | 2nd degree relative | No |
| MEGS_6 | P | Male | 6-10 | 1 to 2 | - | 5 | - | - | Speech/stutter therapy: (unknown method) + to learn speech techniques; Cognitive therapy | 2nd degree relative | No |
| MEGS_7 | P | Female | 6-10 | 3 | - | 5 | - | - | Speech/stutter therapy: (unknown method) + to learn speech techniques | 2nd degree relative | No |
| MEGS_8 | P | Male | 6-10 | 3 | - | 7 | - | - | Speech/stutter therapy: (unknown method) + to learn speech techniques; | 1st degree relative | No |
| MEGS_9 | P | Male | 6-10 | 3 | - | 4 | - | - | Speech/stutter therapy: to learn speech techniques + to change negative thoughts and feelings + to say what you want to say without avoiding sounds/words/situations | No | No |
| MEGS_10 | P | Male | 6-10 | 5 to 6 | - | 6 | - | - | Speech/stutter therapy (unknown method) | No | No |
| MEGS_11 | P | Male | 11-15 | 3 | 7 | 7 | - | - | Hausdorfer method | No | No |
| MEGS_12 | P | Female | 11-15 | 1 to 2 | 4 | 4 | - | - | Speech/stutter therapy (unknown method) | 2nd degree relative | No |
| MEGS_13 | P | Male | 11-15 | 4 | 6 | 7 | - | - | Lidcombe therapy; Speech/stutter therapy to learn speech techniques | No | No |
| MEGS_14 | P | Male | 11-15 | 4 | 3 | 4 | - | - | Lidcombe therapy; RESTART-DCM | No | Spelling difficulty |
| MEGS_15 | P | Male | 11-15 | 4 | 7 | 7 | - | - | Speech/stutter therapy (unknown method) + to learn speech techniques | 2nd (2x) and 3rd degree relative | No |
| MEGS_16 | P | Male | 11-15 | 7 to 8 | 4 | 4 | - | - | Speech/stutter therapy (unknown method) | 2nd degree relative | No |
| KST_1 | P | Male | - | - | - | - | - | - | - | No | - |
| KST_2 | P | Male | 26-30 | 2 | 6 | - | - | - | - | No | No |
| KST_3 | P | Female | 26-30 | 2 to 3 | 6 | - | - | - | - | No | No |
| KST_4 | P | Female | 46-50 | 7 | 7 | - | - | - | - | No | No |
| KST_5 | P | Male | 26-30 | 5 | 3 | - | - | - | - | 1st degree relative | No |
| KST_6 | P | Female | 26-30 | 5 to 6 | 4 | - | - | - | - | No | Otitis media |
| KST_7 | P | Female | 36-40 | School age | 1 | - | - | - | - | 1st degree relative | No |
| KST_8 | P | Male | 41-45 | 1 to 3 | 5 | - | - | - | - | No | No |
| KST_9 | P | Male | 26-30 | 5 | 7 | - | - | - | - | No | Hay fever |
| KST_10 | P | Male | - | - | - | - | - | - | - | No | - |
| KST_11 | P | Male | - | - | - | - | - | - | - | No | - |
| KST_12 | P | Male | 36-40 | 4 | 5 | - | - | - | - | No | No |

A dash (-) indicates no data available. St: stuttering, classified as P: Persistent and T: transient. ^@^Age in years, in age ranges (1-5; 6-10;11-15; etc). ^#^numbers are on scale of 1 to 10, where 1 indicates no stuttering, and 10 indicates very severe stuttering (he/she can hardly produce a word).

**Supplemental Table 2: Enrichment analysis in developmental brain gene expression modules.**

| Module | Gene ontology terms enriched in module | #Control genes | Stuttering genes | P-value | Odds ratio (95%CI) |
| --- | --- | --- | --- | --- | --- |
| M1 | GO:0044425: membrane part  GO:0097458: neuron part  GO:0044456: synapse part | 528 | SPTBN1, ZBTB7A | 0.24 | 2.4 (0.2-11.5) |
| M2 | GO:0019752: carboxylic acid metabolic process  GO:0051186: cofactor metabolic proces | 339 |  | 1.00 |  |
| M3 | GO:0003676: nucleic acid binding  GO:0006325: chromatin organization | 623 | PRPF8, TRIO | 0.31 | 2.0 (0.2-9.6) |
| M4 | GO:0090304: nucleic acid metabolic process  GO:0006613: cotranslational protein targeting to membrane | 460 |  | 1.00 |  |
| M5 | GO:0090304: nucleic acid metabolic process  GO:0003723: RNA binding | 462 |  | 1.00 |  |
| M6 | - | 246 |  | 1.00 |  |
| M7 | GO:0032502: developmental process  GO:0009653: anatomical structure morphogenesis | 259 | ZBTB20 | 0.38 | 2.3 (0.1-16.1) |
| M8 | GO:0006091: generation of precursor metabolites and energy  GO:0044429: mitochondrial part | 170 |  | 1.00 |  |
| M9 | GO:0003676: nucleic acid binding  GO:0010468: regulation of gene expression | 372 | AP4E1 | 0.50 | 1.6 (0.0-11.0) |
| M10 | GO:0031012: extracellular matrix  GO:0001525: angiogenesis  GO:2000145: regulation of cell motility | 189 |  | 1.00 |  |
| M11 | GO:0022402: cell cycle process  GO:0006259: DNA metabolic process | 204 |  | 1.00 |  |
| M12 | GO:0003700: DNA-binding transcription factor activity | 256 | GNPTAB, IREB2 | 0.07 | 5.1 (0.5-25.0) |
| M13 | GO:0044281: small molecule metabolic process | 104 |  | 1.00 |  |
| M14 | GO:0050804: modulation of chemical synaptic transmission  GO:0050773: regulation of dendrite development | 194 |  | 1.00 |  |
| M15 | GO:1990904: ribonucleoprotein complex  GO:0003723: RNA binding | 119 | PPID | 0.19 | 5.1 (0.1-36.3) |
| M16 | GO:0007399: nervous system development  M5GO:0007156: homophilic cell adhesion via plasma membrane adhesion molecules | 141 |  | 1.00 |  |

Only a small set of representative Gene Ontology processes is included.
